# Analysis of influencing factors of perioperative myasthenic crisis in 387 myasthenia gravis patients without thymoma in a single center

**DOI:** 10.1186/s13019-023-02136-1

**Published:** 2023-01-12

**Authors:** Peng Jiao, Fanjuan Wu, Yuxing Liu, Jiangyu Wu, Yaoguang Sun, Wenxin Tian, Hanbo Yu, Chuan Huang, Donghang Li, Qingjun Wu, Chao Ma, Hongfeng Tong

**Affiliations:** 1 Department of Thoracic Surgery, Beijing Hospital, National Center of Gerontology, Institute of Geriatric Medicine, Chinese Academy of Medical Sciences, Beijing, People’s Republic of China; 2 Department of Medicine, Peking University, Beijing, China

**Keywords:** Myasthenia gravis, Postoperative Myasthenic crisis, Thymectomy, ASA score

## Abstract

**Objective:**

To study the influencing factors of myasthenic crisis in non-thymoma myasthenia gravis (MG) patients during perioperative period.

**Methods:**

We retrospectively analyzed a total of 387 non-thymoma MG patients who underwent extended thymoma resection in the Department of Thoracic Surgery of Beijing Hospital from February 2011 to December 2021, recorded ASA score, Osserman classification, preoperative course, pyridostigmine dosage, operation method, operation time, and intraoperative blood loss, then analyzed the factors associated with postoperative myasthenic crisis by univariate and multivariate logistic regression.

**Results:**

Osserman classification IIB + III + IV (*P* < 0.001), history of myasthenic crisis (*P* = 0.013), pyridostigmine dosage greater than 240 (*P* < 0.001), ASA score 2 and 3 (*P* = 0.001) are independent risk factors for myasthenic crisis.

**Conclusion:**

Patients with poor Osserman classification, history of myasthenic crisis before surgery, larger preoperative dosage of pyridostigmine, and higher ASA scores should be highly alert to the occurrence of postoperative myasthenic crisis.

## Introduction

Myasthenia gravis (MG) is an autoimmune disease involving the neuromuscular junction. In 1672 Willis first described the clinical manifestations of MG patients, and in 1912 Saubruch found that thymectomy significantly improved the MG symptoms [1]. With the deepening understanding of myasthenia gravis, thymectomy has become an important part of the treatment. Five years after thymectomy, the complete stable remission rate of MG symptoms can reach more than 50% [2]. A prospective study [3] in 2016 confirmed with a higher level of evidence that MG patients could significantly benefit from thymectomy. Myasthenic crisis (MC) is the most dangerous complication of thymectomy, which can often significantly prolong the hospital stay, increase medical costs and even the risk of death. As the incidence of myasthenia gravis is very low (4.4–6.1/1 million) [4], there are few single-center studies with a large sample size, and it is difficult to unify the all kinds of criteria in meta-analysis with a large sample size [5]. It is also difficult to unify the standards of operation and perioperative management in multi-center studies. Due to the special MG ward in our hospital, there are many such patients, so comprehensive and massive data can be obtained, and the evaluation criteria of each patient are unified. We conducted this retrospective study in order to find out the risk factors of POMC, reduce the occurrence of POMC, or give early warning to avoid more serious complications caused by POMC.

## Methods

Inclusion and Exclusion Criteria:

1. Inclusion Criteria:
  A total of 696 MG patients who was underwent thymectomy in Department of Thoracic Surgery, Beijing Hospital from February 2011 to December 2021.
2. Exclusion Criteria:
  - The patients without MG.
  - The patients with thymoma.

The surgical method was extended thymus resection, which included the whole thymus, the bilateral mediastinal fat and diaphragmatic angle fat. The en bloc resection was performed on the vast majority of patients, and the left and right thymus were removed separately only when the amount of tissue was extremely large and seriously obstructed the surgical field.

POMC should meet the following conditions: within one month after the operation, the patient needs endotracheal intubation or non-invasive ventilator assisted breathing for more than 24 h, or after the endotracheal intubation is removed but because of breathing weakness or inability to cough up intubated again.

All ASA scores were obtained by the same investigator excluding MG.

We investigated the preoperative, intraoperative and postoperative data. The preoperative data included: gender, age, other autoimmune diseases, history of myasthenic crisis, preoperative MG course, Osserman classification, preoperative pyridostigmine bromide dosage, immunosuppressants and glucocorticoid, comorbidities, ASA score, pulmonary function. The intraoperative data included: surgical methods, operation time, intraoperative blood loss. The postoperative data included: drainage volume over the first 3 days after operation, postoperative hospital stay, myasthenic crisis.

Statistical analyses were performed using IBM SPSS version 26.0 statistical software for Windows. All continuous data were presented as mean ± standard deviation and categorical variables were described using frequencies and percentage. The logistic regression analysis was applied for univariate and multivariate analysis to explore risk factors for POMC. Clinical factors that were found to have *p* values of < 0.10 in the univariate analysis were included in a stepwise multiple logistic regression analysis. The results were presented as odds ratios, 95% confidence intervals (CIs) and *p* value. All statistical tests were two-sided and *P* values < 0.05 was considered statistically significant.

## Results

A total of 387 MG patients without thymoma (204 men, 183 women) were included in this study. The mean age was 45.9 years old (15–86 years). POMC occurred in 45 patients (11.6%).

The data of MG patients without thymoma are included as variables in logistic regression analysis in Table 1.

**Table 1:** Basic data

Variables	Data of all patients
n	387
Gender(male/female)	204/183
Age (years old)	45.9 ± 16.3
Other autoimmune disorders (no/yes)	345/42
Preoperative history of myasthenic crisis (no/yes)	380/7
Preoperative course (< 12 months/ ≥ 12 months)	227/160
*Osserman stage*	
I	94 (24.3%)
IIA	126(32.6%)
IIB	135 (34.9%)
III	11 (2.8%)
IV	21 (5.4%)
Preoperative daily dose of pyridostigmine bromide (≤ 240 mg/ > 240 mg)	309/78
Immunosuppressants (no/yes)	337/50
Steroid (no/yes)	337/50
*ASA score*	
1	198 (51.2%)
2	147 (38.0%)
3	42 (10.9%)
FEV1	2.6 ± 0.7
FEV1/FVC	81.4 ± 8.9
FEV1%	83.9 ± 18.4
MVV%	80.2 ± 22.3
DLCO%	90.2 ± 19.4
Operation time (minutes)	128.0 ± 41.8
*Surgical procedure (n[%])*	
OT	28 (7.2%)
VAST	359 (92.8%)
Intraoperative blood loss (0–1000 ml/ > 1000 ml)	383/4
Drainage volume over the first three days (0–800/ > 800)	279/108
Ectopic thymus (yes/no)	365/22
Preoperative application of gamma globulin (yes/no)	352/35

Logistic regression analysis is shown in Table 2. Univariate analysis showed age (*P* = 0.020), preoperative history of myasthenic crisis (*P* < 0.001), Osserman-stage IIB + III + IV (*P* < 0.001), preoperative daily dose of pyridostigmine bromide ≥ 240 mg (*P *< 0.001), ASA grade 2 and 3 (*P *= 0.001), low FEV1 (*P *< 0.001), low FEV1% (*P *= 0.001), low MVV% (*P *= 0.046), low DLCO% (*P *= 0.033), intraoperative blood loss > 1000 ml (*P *= 0.041), preoperative application of gamma globulin (*P *< 0.001) were risk factors for POMC.

**Table 2:** Logistic regression analysis

Variables	Univariate analysis			Multivariate analysis		
	OR	95% CI	*P* value	OR	95% CI	*P* value
*Gender*						
Female	Reference					
Male	1.029	0.552–1.918	0.929			
Age	1.024	1.004–1.045	**0.020**	0.989	0.956–1.024	0.536
Other autoimmune disorders	0.780	0.265–2.300	0.653			
Preoperative history of myasthenic crisis	21.250	3.991–113.136	**0.000**	19.823	1.892–207.705	**0.013**
*Preoperative course*						
< 12 months	Reference			Reference		
≥ 12 months	1.564	0.839–2.917	0.159			
*Osserman classification*						
I + IIA	Reference			Reference		
IIB + III + IV	37.798	9.002–158.709	**0.000**	29.575	5.981–146.237	**0.000**
*Preoperative daily dose of pyridostigmine bromide*						
≤ 240 mg	Reference			Reference		
> 240 mg	6.816	3.534–13.147	**0.000**	6.897	2.544–18.693	**0.000**
Preoperative immunosuppressants	1.042	0.417–2.604	0.930			
Preoperative steroid	1.835	0.825–4.085	0.137			
*ASA score*						
1	Reference			Reference		
2 + 3	3.279	1.638–6.564	**0.001**	8.884	2.526–31.246	**0.001**
FEV1	0.327	0.189–0.566	**0.000**	0.506	0.152–1.679	0.266
FEV1/FVC	0.988	0.951–1.026	0.531			
FEV1%	0.971	0.954–0.988	**0.001**	0.995	0.961–1.030	0.767
MVV%	0.983	0.966–1.000	**0.046**	1.006	0.985–1.028	0.557
DLCO%	0.980	0.963–0.998	**0.033**	0.980	0.957–1.005	0.116
Operation time	1.005	0.998–1.012	0.153			
*Surgical procedure*						
OT	Reference			Reference		
VAST	1.293	0.427–3.912	0.650			
*Blood loss*						
0–1000	Reference			Reference		
> 1000	7.907	1.086–57.582	**0.041**	27.550	0.926–819.913	0.055
*Drainage volume over the first three days*						
0–800	Reference					
> 800	1.191	0.607–2.338	0.611			
Ectopic thymus	0.749	0.169–3.316	0.703			
Preoperative application of gamma globulin	5.909	2.720–12.839	**0.000**	1.179	0.393–3.531	0.769

Bold value indicates the *P*-value is lower than 0.05

*FEV1* Forced expiratory volume in 1 s, *FVC* Forced vital capacity, *MVV* Maximal voluntary ventilation, *DLCO* Diffusion lung capacity for carbon monoxide, *OT* Open thoracotomy, *VAST* Video-assisted thoracic surgery

Variables that showed a *P* value < 0.10 in univariate logistic regression analysis were entered into a multivariate logistic regression analysis. The result showed that preoperative history of myasthenic crisis (*P *= 0.013), Osserman-stage IIB + III + IV (*P *< 0.001), preoperative daily dose of pyridostigmine bromide ≥ 240 mg (*P *< 0.001), ASA grade 2 and 3 (*P *= 0.001) were independent risk factors for POMC.

## Discussion

The incidence of POMC in non-thymoma MG in our study was 11.63%, which is lower than previous studies [5–8]. Osserman type IIb and above are independent risk factors for our POMC (*P *< 0.001), which is also a relatively well recognized result [5, 9–13]. These patients have muscle weakness in swallowing, chewing, articulation, and breathing because of the involvement of the bulbar-related muscle groups. In addition to inspiratory and expiratory muscles, facial, oropharyngeal, and laryngeal muscles are important to maintain respiratory function. Weakness in these muscles interferes with upper airway protection, swallowing, and secretion clearance, even lead to upper airway obstruction and respiratory failure [14]. In addition to the above reasons, clinically, we conclude that most of these symptoms are caused by intraoperative tracheal intubation stimulation, pyridostigmine bromide and other reasons that lead to more airway secretions and inability to cough up, plus the saliva is unable to swallow or spit out, which is easy to cause aspiration or blockage of airway. POMC can often not be avoided, even we adjust the dosage of pyridostigmine bromide and anisodamine in real time according to the patient's condition. In order to reduce the airway irritation caused by intraoperative tracheal intubation, almost all the surgical methods have been changed to thoracoscopic thymectomy through the subxiphoid approach in the past two years, enabling us to perform laryngeal mask-assisted ventilation instead of tracheal intubation. However, at present, the amount of data is not enough, and there are many other influencing factors, and the protective advantage to POMC has not yet been shown.

Our multivariate analysis showed that a history of MC was a predictive factors for POMC(*P *= 0.013), whereas preoperative MG severity was not, which was also mentioned in some other studies [5]. About 20% of MG patients develop MC in their lifetime and one-third of them have a second MC [15]. This means that if the patient has ever had severe MG symptoms, although the symptoms are well controlled before surgery, MC may still occur after surgery, anesthesia and other blows. Therefore, even if the symptoms of these patients are well controlled, they should not be taken lightly. Postoperative management needs to be strengthened to be alert to the occurrence of POMC.

Another independent risk factor for POMC was the preoperative dose of 240 mg or more of pyridostigmine bromide daily (*P* < 0.001). Other studies [5] may not be exactly 240 mg, but in general, POMC is more likely to occur at higher dose. Acetylcholinesterase inhibitor (AChEI) increases the concentration of acetylcholine by blocking acetylcholinesterase at the neuromuscular junction to improve muscle strength. The large doses of cholinesterase inhibitors will accelerate the destruction of postsynaptic acetylcholine receptors (AChRs) at the neuromuscular junction, which decreases the postoperative ability of patients to cough up sputum, causes excessive respiratory secretions, increase the risk of respiratory system infection. The above factors will increase the risk of POMC. On the other hand, the larger dosage reflects severer symptoms. Therefore, the dosage of pyridostigmine bromide should be adjusted to the minimum dose range that can control symptoms before surgery as far as possible.

In the past, it seems that no one regarded preoperative comorbidities and basic health status of patients as one of the evaluation indicators of POMC. Clinically, we found that patients with poor preoperative general condition may be more prone to POMC. Therefore, we used the American Society of Anesthesiologists (ASA) (2020 edition) system to assess the preoperative comorbidities and underlying health status of patients. ASA classification is designed primarily to predict and evaluate the risk of anesthesia in surgery, which was first proposed in 1941 [16]. It has been verified by a large number of clinical cases and modified for many times. The ASA score system is also significantly correlated with the incidence of postoperative complications in different surgical specialties [17–20]. In our study, we found that ASA score was an independent influencing factor of POMC (*P* = 0.001), indicating that patients with more preoperative comorbidities were more likely to develop POMC. ASA as a predictor of POMC has never been mentioned in previous studies and needs to be further validated.

In our study, 35 patients with Osserman classification IIb or above, and unsatisfactory MG symptom control, received preoperative gamma globulin. As a high-risk group, 13 of them achieved POMC, with an incidence rate of 37.14%. Many studies [21] believe that gamma globulin can reduce the incidence of POMC. In our univariate analysis, preoperative gamma globulin was a possible risk factor, but in multivariate analysis, it did not become an independent risk factor. However, Gamez et al. conducted a prospective randomized controlled study in 2019 [22], suggesting that the application of gamma globulin could not reduce the risk of postoperative POMC in patients with well-controlled preoperative MG symptoms.

It must be acknowledged that there are some deficiencies in this study: 1. This is a retrospective study; 2. Limited to conditions, many patients did not have MG-related antibody tests, and many patients only had AChR-Ab, but no MuSK-Ab and other tests; 3. Preoperative treatment is inconsistent, and some of the high-risk patients with POMC were treated with gamma globulin, which inevitably affected the results; 4. Some patients are unable to cooperate with the pulmonary function test before surgery, so the result of the pulmonary function may not be accurate.

Through the study of perioperative factors in 387 non-thymoma MG patients, we concluded that the independent risk factors for POMC were: history of previous crisis, preoperative MG Osserman stage IIb and or above, preoperative dose of pyridoxine bromide ≥ 240 mg, and poor ASA classification. Therefore, non-thymoma MG patients should be highly vigilant if combined with the above conditions, take countermeasures in advance, and timely deal with the MG crisis that may occur at any time to ensure the safety of patients.

## Data Availability

The datasets generated during and/or analysed during the current study are available from the corresponding author on reasonable request.
